# Food spending in the United States during the first year of the COVID-19 pandemic

**DOI:** 10.3389/fpubh.2022.912922

**Published:** 2022-08-03

**Authors:** Chandra Dhakal, Binod Acharya, Shaonan Wang

**Affiliations:** ^1^Department of Agricultural and Applied Economics, University of Georgia, Athens, GA, United States; ^2^Urban Health Collaborative, Drexel University, Philadelphia, PA, United States

**Keywords:** food spending, food security, COVID-19, pandemic, consumer expenditure survey

## Abstract

The COVID-19 pandemic brought about a significant increase in the unemployment rate and a decline in consumer income. At the same time, the public health responses to the pandemic, such as lockdowns and business closures, disrupted the food supply chain. These pandemic-driven changes could lead to a shift in food spending behaviors and potentially exacerbate the food insecurity situation. Leveraging the nationally representative dataset from the 2017–2020 consumer expenditure surveys, we employ a two-part model to assess the changes in weekly household spending on total food, food-at-home (FAH), and food-away-from-home (FAFH) between the pre-pandemic and pandemic period in the United States. Our finding shows a predicted marginal decline in FAFH expenditure by 33.7% but an increase in FAH spending by 6.9% during the pandemic. The increase in FAH spending could not fully offset the decrease in FAFH spending, leading to a decline in total food spending by 12.6%. The results could provide a basis for future studies on food insecurity, nutrient intake, and healthy consumption during the pandemic.

## Introduction

The COVID-19 pandemic impacted every domain of life, including consumer expenditure decisions. It threatened global food security (1) and created a shock to the food and agricultural system in the US (2). Unprecedented unemployment rates and recession pushed millions of Americans into financial hardship (3, 4). A decline in household income during the period of the economic crisis may alter the consumers' attribution of “food values” (5) and purchase decisions (6). For example, rising unemployment is associated with a reduced intake of fruits and vegetables and increased consumption of “unhealthy” fast foods (7). Moreover, the supply-chain disruption due to stay-at-home orders, business closures, and social distancing mandates during the pandemic forced many restaurants to close or operate in a limited capacity. These factors, in conjunction with consumers' voluntary avoidance of dining out because of the perceived risk of virus infection and the rise of online grocery shopping, may have affected food acquisition behaviors, making food-at-home (FAH) a more preferred choice over food-away-from-home (FAFH) (8).

Historical spending data from the US suggests that expenditure on FAH and FAFH increased from 1997 to 2019, with the real FAH increasing at a slower rate (39.7%) than FAFH (60.5%) (9). Since 2010, the spending on FAFH has surpassed spending on FAH. The higher share of FAFH in total food spending is attributed in part to the lesser available time for meal preparation at home (10). However, the high unemployment rates in the pandemic may have increased the available time for cooking, which could increase the share of FAH spending (10). A few studies utilizing small sample sizes and a narrower study window have indicated a pandemic-driven increase in FAH and a decrease in FAFH (8, 11). These studies do not fully explain to what extent the food expenditure decisions changed in the entire US and whether the substitution of FAFH by FAH is sufficient to maintain the household's food security.

There are concerns of worsening food security due to the COVID-19 pandemic in the US, where food insecurity remains one of the leading health and nutrient issues (12, 13). Studies indicate that ~10–15 % of the US households experienced food insecurity early in the pandemic (14, 15), with millions of Americans affected (16–18) and that the total food spending in the first year of the pandemic decreased (19). The decline in total food spending could likely translate into reduced calorie intake or poor quality food consumption, compounding consumer health risks and food security situations (20, 21). Moreover, the pandemic may have changed the attitudinal and behavioral views of consumers about foods, as indicated by lowered eating competence (22). The individual consumption pattern was also affected by the epidemic (23). Households, particularly low-income households, have become more sensitive to food prices as a result of price shocks from food supply chain disruptions and income shocks from unemployment (24, 25). Self-quarantine and mobility restrictions changed households' choices of transportation means (26) and prompted them to increase online shopping (27). Although a few studies have explored the changes in food expenditure and food insecurity in relation to the pandemic (8, 28) they are narrower in scope because of the limited time frame, geography, and smaller sample size. We aim to fill that gap in the literature by analyzing the nationally representative data for 3 years. Our study uses high-frequency data to well-capture the changes in household food consumption during the pandemic. In particular, the objective of this paper is to examine the changes in weekly US household food spending during the COVID-19 pandemic compared to the pre-pandemic period. To better understand the effects of the pandemic on household food spending dynamics, we separately examine the expenditure for total Food, FAH, and FAFH. Meanwhile, the nationally representative data allows us to control for the household heterogeneity. Due to the restricted transportation means, households are more likely to make purchases less frequently but in larger quantities at one purchase. Therefore, they may not purchase food in a given week. Our study takes into account the estimation bias caused by the censoring of purchases using the two-part model, and gives us a consistent estimator.

## Methods

### Study sample and measures

We obtained the public use microdata of weekly household food expenditures (total, FAFH, and FAH) from the Consumer Expenditure (CE) Diary Survey, conducted by the US Census Bureau for the Bureau of Labor Statistics (BLS). The CE survey collects information on frequently purchased items *via* the Diary Survey, a cross-sectional survey requiring households to report 1-week expenditures for two consecutive weeks. The Diary survey consists of two short in-person interviews about 2-weeks apart and records purchases made by everyone who lives in the household (29). We analyzed the data between Jan 2017 and Dec 2020, where we defined Jan 2017–Feb 2020 as the “pre-pandemic” and Mar 2020–Dec 2020 as the “pandemic” period. We used dollars spent on total food, FAFH, and FAH weekly purchases as outcome variables. The indicator “pandemic (1/0)” is the primary exposure variable of interest.

### Statistical analysis

Because not all households reported food purchases during the survey week, there were many zero expenditures in the data. We utilized a two-part modeling strategy (30, 31) to address this issue. We applied the logit model for the first part to model the probability of food purchase and multiple linear regression for the second part to estimate the expected amount of expenditure (log) whenever there is a purchase. The covariates in the model were: age, gender, race/ethnicity, annual household income, education, family size, and indicator for food stamps participation in the past 12 months. We controlled our analysis for the month of the survey response, the US census region, and the urban/rural indicator. We fit separate models for total Food, FAFH, and FAH spending. The analysis incorporated the survey weights provided by BLS. Standard errors were clustered at the state level. The two-part modeling was implemented in Stata 17, using the *twopm* command (31). We calculated the marginal predicted expenditure by combining the results from both parts, with the command: *margins pandemic, predict (duan) post*.

## Results

The weighted distribution of survey respondents (unweighted *N* = 38,080 household-week) and their corresponding total food, FAFH, and FAH expenditure are presented in Table 1. High-income and more educated households spent more on food, whereas low-income, less educated, and food stamps participating households spent less. Food expenses by households led by Non-Hispanic Blacks were lower compared to Non-Hispanic Whites or Hispanics. The average household food expenditure during the study window was $149.2, where more than 38% of spending was for FAFH. The share of the FAFH in total food spending during the pre-pandemic period was 40.8% but it was only 28.8% during the pandemic (Table 1). Conversely, the share of FAH increased during the pandemic (59.1%) compared to the pre-pandemic period (71.2%).

**Table 1:** Characteristics of respondents and the corresponding weekly food spending (unweighted N = 38,080 household-weeks).

**Characteristics**	**% of respondents**	**Total food spending dollars per week** **(Mean, SD)**			**FAFH spending dollars per week** **(Mean, SD)**			**FAH spending dollars per week** **(Mean, SD)**		
		**4-year**	**Pre-pandemic**	**Pandemic**	**4-year**	**Pre-pandemic**	**Pandemic**	**4-year**	**Pre-pandemic**	**Pandemic**
**Age (years)**										
16–30	14.7	116.0 (107.0)	116.3 ( 103.0)	114.7 (124.8)	52.4 (66.1)	54.4 (65.9)	42.6 (66.2)	63.6 (74.9)	61.9 (70.5)	72.1 (93.5)
31–45	25.9	172.8 (155.1)	174.6 (154.7)	166.5 (156.4)	68.6 (90.8)	73.8 (94.8)	50.2 (72.4)	104.2 (110.1)	100.8 (104.3)	116.3 (127.9)
46–55	17.9	176.6 (156.3)	179.4 (156.9)	166.3 (153.5)	69.2 (92.1)	74.2 (95.0)	50.9 (77.8)	107.4 (105.8)	105.2 (101.9)	115.5 (118.7)
56–65	18.7	149.1 (150.8)	152.1 (151.2)	138.1 (149.0)	51.4 (88.7)	56.5 (95.7)	33.1 (52.3)	97.7 (108.1)	95.7 (102.7)	105.0 (125.1)
65 plus	22.7	122.1 (180.7)	125.2 (187.6)	111.8 (155.0)	42.8 (150.0)	47.1 (162.8)	28.3 (93.9)	79.3 (87.4)	78.1 (78.4)	83.6 (112.3)
**Gender**										
Male	45.5	155.6 (151.2)	158.1 (150.8)	146.0 (152.5)	62.6 (95.7)	67.4 (97.6)	44.1 (85.8)	93.0 (102.8)	90.7 (99.5)	101.9 (114.2)
Female	54.5	143.8 (161.1)	145.4 (163.3)	138.0 (152.6)	52.8 (111.8)	56.8 (121.0)	38.1 (67.0)	91.0 (98.9)	88.6 (90.7)	99.9 (123.6)
**Race/ethnicity**										
Non-hispanic white	64.7	155.9 (161.7)	158.0 (163.3)	148.4 (155.6)	60.3 (112.1)	64.9 (118.7)	43.5 (81.7)	95.6 (100.7)	93.1 (95.5)	104.9 (117.4)
Non-hispanic black	13.5	108.9 (121.0)	108.2 (109.7)	111.2 (156.7)	38.6 (61.3)	41.5 (63.5)	27.9 (51.0)	70.2 (95.8)	66.8 (77.2)	83.3 (145.7)
Hispanic	14.5	144.9 (144.0)	148.8 (147.3)	130.3 (129.7)	55.2 (81.5)	59.8 (84.3)	37.9 (67.3)	89.7 (101.1)	88.9 (101.5)	92.4 (99.6)
Other	7.2	172.9 (181.0)	176.8 (187.8)	158.0 (151.3)	68.7 (135.5)	74.8 (146.7)	45.6 (75.1)	104.2 (102.3)	102.0 (98.0)	112.4 (116.9)
**Food stamps in the past 12 months**										
Received	9.3	105.5 (126.1)	106.5 (120.9)	101.9 (142.3)	27.4 (57.0)	30.3 (61.4)	17.7 (36.6)	78.1 (9.1)	76.3 (88.7)	84.2 (128.0)
Not received	90.7	154.0 (159.9)	156.4 (161.8)	145.2 (152.3)	60.9 (109.5)	65.5 (115.8)	43.6 (79.0)	93.1 (100.6)	90.9 (95.6)	101.5 (117.3)
**Education**										
Less than high school	8.5	117.7 (150.0)	118.0 (150.4)	116.3 (148.5)	36.1 (73.4)	38.2 (77.8)	26.2 (47.6)	81.6 (111.6)	79.8 (106.1)	90.1 (133.9)
High school graduate	41.9	128.1 (152.5)	131.0 (156.9)	116.8 (133.5)	47.8 (111.1)	51.5 (121.1)	33.3 (55.7)	80.3 (90.9)	79.5 (84.7)	83.5 (111.8)
Bachelor's degree	34.8	163.9 (151.0)	166.4 (147.6)	154.8 (162.2)	65.7 (96.1)	70.9 (95.9)	46.7 (94.5)	98.2 (102.0)	95.5 (97.1) _	108.1 (117.3)
Above bachelor's degree	14.8	192.0 (172)	194.7 (174.5)	183.4 (163.4)	76.2 (116.2)	83.9 (124.5)	52.4 (81.3)	115.8 (111.1)	110.9 (104.8)	131.0 (127.8)
**Annual household income**										
< $25,000	20.5	83.6 (110.6)	84.3 (108.4)	80.6 (119.8)	26.7 (66.9)	28.9 (71.2)	17.6 (42.8)	56.9 (80.8)	55.4 (73.3)	63.0 (106.6)
$25,000–$50,000	22.9	116.3 (115.1)	119.1 (108.6)	106.2 (136.0)	41.2 (70.9)	44.3 (64.3)	29.6 (90.4)	75.2 (80.7)	74.8 (78.1)	76.6 (89.6)
$50,000–$100,000	27.9	147.8 (166.1)	152.4 (175.0)	130.4 (125.1)	57.3 (130.1)	62.4 (142.8)	37.5 (55.9)	90.5 (93.4)	89.9 (89.9)	92.8 (105.8)
$100,000–$15,0000	13.9	194.9 (147.3)	197.0 (146.7)	187.1 (149.6)	77.0 (91.0)	82.9 (94.7)	55.4 (71.7)	117.9 (107.3)	114.1 (102.3)	131.6 (122.9)
>$15,0000	14.8	250.2 (191.0)	256.4 (191.2)	232.3 (189.9)	105.8 (127.7)	117.0 (134.7)	72.8 (97.2)	144.4 (129.1)	139.3 (119.7)	159.4 (152.6)
**Overall**	100	149.2 (156.8)	151.2 (157.8)	141.6 (152.6)	57.2 (104.9)	61.7 (111.0)	40.8 (76.0)	91.9 (100.7)	89.5 (94.8)	100.7 (119.5)

FAFH and FAH represent food-away-from-home, and food-at-home, respectively, and SD is the standard deviation. Numbers incorporate survey weights.

We present the regression coefficients from two-part models in Table 2. The coefficients from the first part are in the logit scale, and exponentiating them results in the Odds Ratio (OR) estimates of making purchases associated with a given variable. The ORs of total food, FAFH, and FAH spending associated with the pandemic were 0.31 (95% CI: 0.26, 0.37), 0.38 (CI: 0.34, 0.42), and 0.58 (CI: 0.49, 0.67), respectively, indicating that people were significantly less likely to make food purchases during the pandemic. The coefficients from the second part denote the changes in expenditure amount associated with a given variable, conditional on positive spending. Since the outcome variable was log-transformed, we estimate that a unit change (or compared to the reference group) in the predictor variable is associated with (*e*^β^−1) × 100% change in expenditure. We found a decrease of 8.6% (CI: 3.9 12.2) in total food spending during the pandemic. Compared to the pre-pandemic, the conditional spending on FAFH decreased by 16.5% (CI: 12.2, 20.5), while it increased for FAH by 13.9% (CI: 9.4, 18.5) during the pandemic.

**Table 2:** Regression coefficient (95% Confidence Interval) from two-part models.

**Variables**	**Part 1: Logit on expenditure vs. no** **expenditure**			**Part 2: Multiple linear regression on log** **(expenditure) conditional on positive expenditure**		
	**Total food**	**FAFH**	**FAH**	**Total food**	**FAFH**	**FAH**
Pandemic (Ref: Pre-pandemic)	−1.17*** (−1.34, −1.00)	−0.97*** (−1.09, −0.86)	−0.55*** (−0.71, −0.40)	−0.09*** (−0.13, −0.04)	−0.18*** (−0.23, −0.13)	0.13*** (0.09, 0.17)
**Age, years (Ref:16–30)**						
31–45	−0.12 (−0.31, 0.07)	−0.24*** (−0.36, −0.13)	0.37*** (0.22, 0.51)	0.14*** (0.10, 0.18)	0.02 (−0.03, 0.08)	0.19*** (0.15, 0.23)
46–55	0.11 (−0.15, 0.37)	−0.27*** (−0.40, −0.14)	0.66*** (0.54, 0.77)	0.17*** (0.12, 0.22)	−0.00 (−0.07, 0.06)	0.26*** (0.22, 0.30)
56–65	0.01 (−0.20, 0.21)	−0.50*** (−0.64, −0.37)	0.87*** (0.75, 1.00)	0.19*** (0.15, 0.23)	−0.13*** (−0.19, −0.06)	0.32*** (0.28, 0.37)
65 plus	−0.11 (−0.30, 0.07)	−0.72*** (−0.84, −0.60)	0.89*** (0.76, 1.02)	0.13*** (0.08, 0.18)	−0.13*** (−0.19, −0.06)	0.28*** (0.23, 0.33)
Female (Ref: male)	0.01 (−0.14, 0.16)	−0.10** (−0.19, −0.02)	0.14*** (0.06, 0.22)	−0.04*** (−0.06, −0.01)	−0.08*** (−0.11, −0.04)	0.00 (−0.02, 0.03)
**Race/ethnicity (Ref: hispanic)**						
Non–hispanic white	−0.11 (−0.30, 0.08)	0.11 (−0.12, 0.35)	−0.02 (−0.17, 0.12)	0.03 (−0.01, 0.07)	−0.04 (−0.13, 0.05)	0.07* (−0.00, 0.14)
Non–hispanic black	−0.32 (−0.72, 0.08)	−0.24** (−0.48, −0.00)	−0.29*** (−0.44, −0.15)	−0.24*** (−0.31, −0.18)	−0.24*** (−0.37, −0.10)	−0.18*** (−0.26, −0.11)
Others	0.03 (−0.34, 0.41)	−0.16 (−0.38, 0.07)	−0.01 (−0.20, 0.19)	−0.02 (−0.08, 0.04)	−0.12* (−0.24, 0.00)	0.07 (−0.02, 0.15)
**Annual household income (Ref:** ** < $25,000)**						
$25,000–$50,000	0.21*** (0.09, 0.32)	0.46*** (0.35, 0.57)	0.11** (0.01, 0.22)	0.29*** (0.26, 0.33)	0.21*** (0.15, 0.28)	0.20*** (0.16, 0.25)
$50,000–$10,0000	0.39*** (0.26, 0.51)	0.68*** (0.56, 0.80)	0.15** (0.02, 0.27)	0.44*** (0.39, 0.49)	0.38*** (0.33, 0.43)	0.31*** (0.25, 0.38)
$10,0000–$15,0000	0.70*** (0.49, 0.92)	1.02*** (0.90, 1.15)	0.33*** (0.19, 0.47)	0.64*** (0.59, 0.69)	0.60*** (0.54, 0.65)	0.44*** (0.38, 0.51)
>$15,0000	0.54*** (0.29, 0.79)	1.09*** (0.95, 1.24)	0.16** (0.02, 0.30)	0.84*** (0.79, 0.89)	0.89*** (0.82, 0.96)	0.61*** (0.54, 0.67)
**Education (Ref: Less than high school)**						
High schoolgraduate	0.10 (−0.20, 0.41)	0.32*** (0.18, 0.46)	0.05 (−0.10, 0.20)	0.09** (0.02, 0.17)	0.11*** (0.03, 0.20)	0.03 (−0.04, 0.10)
Bachelor's degree	0.51*** (0.23, 0.80)	0.56*** (0.38, 0.73)	0.32*** (0.19, 0.45)	0.24*** (0.17, 0.32)	0.22*** (0.13, 0.31)	0.14*** (0.07, 0.20)
Above bachelor's degree	0.56** (0.08, 1.03)	0.47*** (0.24, 0.70)	0.39*** (0.16, 0.63)	0.27*** (0.18, 0.37)	0.23*** (0.12, 0.34)	0.20*** (0.10, 0.29)
Family size	0.01 (−0.06, 0.09)	−0.01 (−0.04, 0.02)	0.20*** (0.16, 0.24)	0.14*** (0.12, 0.15)	0.06*** (0.04, 0.08)	0.17*** (0.16, 0.18)
Participated in food stamp, No (Ref: Yes)	0.38*** (0.12, 0.63)	0.40*** (0.29, 0.51)	0.09 (−0.10, 0.27)	0.13*** (0.09, 0.18)	0.28*** (0.20, 0.36)	0.05** (0.01, 0.10)
Rural (Ref: Urban)	0.06 (−0.35, 0.47)	−0.22* (−0.44, 0.00)	0.08 (−0.13, 0.29)	−0.03 (−0.17, 0.11)	−0.05 (−0.18, 0.07)	−0.01 (−0.14, 0.13)
**Region (Ref: northeast)**						
Midwest	−0.21* (−0.44, 0.02)	0.25*** (0.10, 0.40)	−0.12 (−0.34, 0.11)	−0.07** (−0.14, −0.00)	−0.04 (−0.14, 0.05)	−0.10*** (−0.18, −0.03)
South	−0.40*** (−0.61, −0.18)	0.14 (−0.04, 0.33)	−0.23** (−0.44, −0.02)	−0.04 (−0.12, 0.03)	0.01 (−0.08, 0.10)	−0.09* (−0.19, 0.00)
West	0.16 (−0.09, 0.41)	0.26** (0.01, 0.51)	0.07 (−0.18, 0.32)	0.02 (−0.06, 0.09)	0.07 (−0.08, 0.22)	−0.05 (−0.13, 0.02)
**Month (Ref: Jan)**						
Feb	0.43* (−0.03, 0.88)	0.07 (−0.05, 0.19)	0.10 (−0.08, 0.29)	0.06 (−0.01, 0.14)	0.11*** (0.04, 0.17)	−0.01 (−0.09, 0.07)
Mar	0.07 (−0.31, 0.45)	0.07 (−0.10, 0.25)	0.01 (−0.20, 0.22)	0.05 (−0.03, 0.13)	0.06* (−0.01, 0.12)	−0.01 (−0.10, 0.07)
Apr	0.03 (−0.33, 0.39)	−0.10 (−0.28, 0.09)	0.07 (−0.13, 0.27)	0.06 (−0.02, 0.14)	0.06 (−0.02, 0.14)	0.02 (−0.04, 0.09)
May	0.02 (−0.30, 0.34)	−0.08 (−0.26, 0.09)	0.10 (−0.05, 0.24)	0.02 (−0.05, 0.09)	0.05 (−0.02, 0.13)	−0.02 (−0.10, 0.06)
Jun	−0.09 (−0.50, 0.32)	0.07 (−0.10, 0.23)	−0.07 (−0.27, 0.12)	0.04 (−0.02, 0.10)	0.08** (0.01, 0.15)	−0.03 (−0.11, 0.05)
Jul	−0.10 (−0.45, 0.26)	0.01 (−0.15, 0.16)	0.02 (−0.16, 0.19)	0.04 (−0.02, 0.10)	0.09** (0.01, 0.16)	−0.02 (−0.09, 0.05)
Aug	−0.08 (−0.45, 0.28)	0.05 (−0.13, 0.22)	−0.01 (−0.17, 0.15)	0.05 (−0.01, 0.10)	0.07** (0.00, 0.15)	−0.02 (−0.08, 0.05)
Sep	−0.20 (−0.64, 0.25)	−0.00 (−0.15, 0.15)	−0.06 (−0.25, 0.13)	0.04 (−0.02, 0.11)	0.11*** (0.05, 0.17)	−0.03 (−0.11, 0.04)
Oct	0.12 (−0.37, 0.60)	0.15* (−0.00, 0.30)	0.02 (−0.20, 0.23)	0.10*** (0.04, 0.16)	0.16*** (0.09, 0.23)	0.02 (−0.03, 0.06)
Nov	−0.06 (−0.47, 0.34)	−0.09 (−0.23, 0.06)	0.01 (−0.20, 0.21)	0.03 (−0.03, 0.09)	0.05** (0.00, 0.10)	0.01 (−0.07, 0.08)
Dec	−0.09 (−0.46, 0.27)	0.01 (−0.14, 0.16)	0.03 (−0.16, 0.21)	0.08** (0.01, 0.15)	0.10*** (0.03, 0.17)	0.02 (−0.05, 0.09)
Constant	2.60*** (1.88, 3.31)	−0.10 (−0.41, 0.22)	0.66*** (0.20, 1.12)	3.36*** (3.26, 3.47)	2.63*** (2.39, 2.86)	3.11*** (2.97, 3.25)

*Significant at the 10% level; **Significant at the 5% level; ***Significant at the 1% level; FAFH and FAH represent food-away-from-home, and food-at-home, respectively.

**Figure 1:**
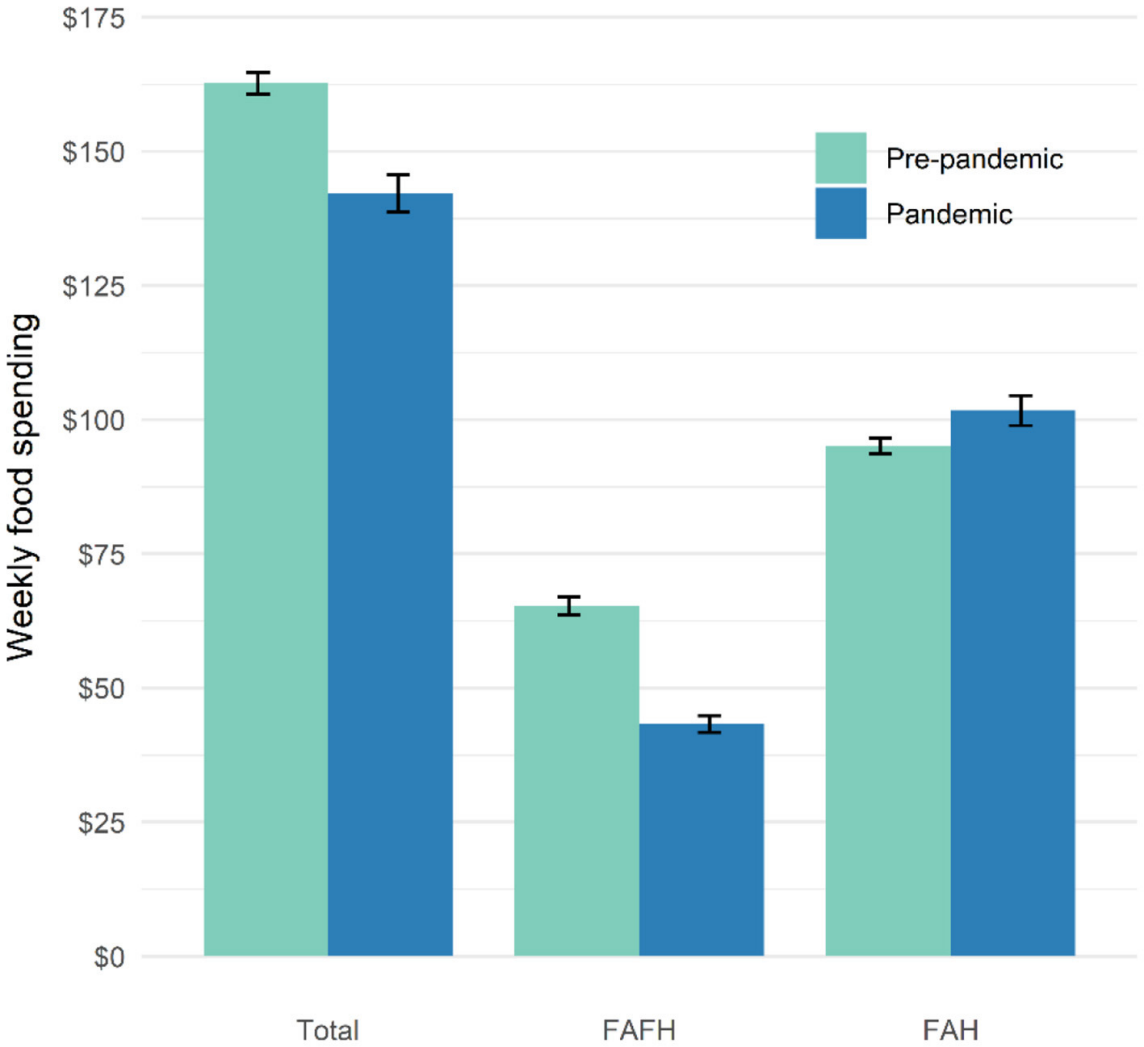
Predicted marginal expenditure from the two-part model. Error bars represent the means ± one standard error from the Delta method.

We calculated the marginal predicted expenditure by combining the results from both parts (Figure 1). We found that the weekly total food dollars decreased from $162.6 to $142.2, corresponding to a 12.6% decrease. The FAFH spending decreased by 33.7%, from $65.3 in the pre-pandemic to $43.3 in the pandemic. In contrast, the FAH increased by 6.9%, from $95.1 to $101.7.

## Discussion

The COVID-19 pandemic affected every aspect of human life, including food spending and eating behaviors. The FAFH spending decreased during the pandemic, while the FAH spending increased. This substitution of FAFH by FAH is perhaps unsurprising given the government-issued mandates that restricted the operation of restaurants, bars, and sporting events as well as public health responses such as stay-at-home orders and social-distancing measures aimed to contain the spread of the virus (32). Even when the FAFH establishments were open, they operated in a limited capacity, and many of them switched to pick-up or delivery-only options, ramping up FAH purchases (8). Furthermore, FAH may have been favored because of the consumers having more available time to prepare the meal at home (e.g., due to reduction in commuting time caused by remote working or the increased availability of leisure time due to unemployment).

The substitution of FAFH by FAH, however, was not sufficient to offset the reduction in FAFH spending, leading to a significant decline in total food expenses. In fact, during the pandemic, household cut their total food spending on average by 12.6%. The magnitude of the decline in food spending during this pandemic is much bigger compared to the 1.5 and 3% decline in food spending observed in 2008 and 2009, respectively (9). Barring the Great Recession period in 2008–2009, food spending had been consistently increasing in the United States between 1997 and 2019 (9). The COVID-19 pandemic has effectively upended this trend, raising concerns about elevated food insecurity.

Although the results are mixed as to what extent the pandemic impacted the expenditure on risky health products such as alcohol and tobacco (33–36), the significant decline in total food spending is concerning since it likely indicates increased food insecurity. Several published studies have indicated higher food insecurity during the pandemic (14, 16, 17), particularly among older and disadvantaged adults (18). Our finding of decreased total food spending, taken together with emerging studies about food security during the pandemic, may lend some evidence that household food security might have worsened during the pandemic.

The pandemic has impacted household preferences for food choices and healthy diets (37, 38). Especially for low-income households, the epidemic has made them more vulnerable, and they are more likely to suffer from food insecurity and nutritional risks. Our finding speaks to the importance of undertaking measures to strengthen the Supplemental Nutrition Assistance Program (SNAP) and Special Supplemental Nutrition Program for Women, Infants, and Children (WIC), which requires sustained and coordinated federal policies (39). Future studies can extend our study to investigate food spending heterogeneity based on different income stratum and examine the change in household preferences for healthy food and unhealthy food during the pandemic. Future research should focus on identifying the mechanism through which the COVID-19 pandemic might affect food spending behavior, including the nutrient content of the food purchased.

The study has several limitations. The CE Diary Survey is a cross-sectional survey, and as such, we lack the flexibility of panel data. Also, we modeled the food expenditure and were not able to consider possible underreporting of expenditure as well as the calorie or quality of food items purchased and consumed. Furthermore, we could not account for the relative price differentials in the FAFH and FAH, which suggests that the decrease in total food spending does not necessarily translate into lower food insecurity to the extent that consumers are paying smaller prices for FAH.

## Conclusion

During the pandemic, the FAFH expenditure decreased, but the spending on FAH increased. The increase in FAH spending was insufficient to offset the decrease in FAFH spending, leading to a net decline in total food expenses. Barring the Great Recession of 2008–2009, the long-term trend of total food spending in the United States was upward. The COVID-19 pandemic reversed this trend with households cutting their expenses on food. This significant decline in food spending raises concerns about the worsening food security situation. Our findings could be valuable in understanding changes in consumer food purchasing behaviors during periods of economic downturn and public health emergencies.

## Data availability statement

Publicly available datasets were analyzed in this study. This data can be found here: (Consumer Expenditure Survey) https://www.bls.gov/cex/.

## Author contributions

CD and BA conceptualized the study and performed the statistical analysis. CD, BA, and SW wrote the first draft, reviewed, and edited the manuscript. All authors contributed to manuscript revision, read, and approved the submitted version.

## Conflict of interest

The authors declare that the research was conducted in the absence of any commercial or financial relationships that could be construed as a potential conflict of interest.

## Publisher's note

All claims expressed in this article are solely those of the authors and do not necessarily represent those of their affiliated organizations, or those of the publisher, the editors and the reviewers. Any product that may be evaluated in this article, or claim that may be made by its manufacturer, is not guaranteed or endorsed by the publisher.
